# Integrative analysis of DNA methylation-driven genes for the prognosis of lung squamous cell carcinoma using MethylMix

**DOI:** 10.7150/ijms.43272

**Published:** 2020-03-05

**Authors:** Rui Li, Yun-Hong Yin, Jia Jin, Xiao Liu, Meng-Yu Zhang, Yi-E Yang, Yi-Qing Qu

**Affiliations:** 1 Department of Respiratory and Critical Care Medicine, Qilu Hospital of Shandong University, Jinan 250012, China.; 2 Department of Cardiology, Zhangqiu District People's Hospital of Jinan, 250200, Shandong, China.; 3 Department of Clinical Laboratory, Shandong Provincial Qianfoshan Hospital, the First Hospital Affiliated with Shandong First Medical University, Jinan 250014, China.

**Keywords:** Lung squamous cell carcinoma, Methylation-driven genes, Biomarkers, A Cox predictive model, Overall survival

## Abstract

**Background**: DNA methylation acts as a key component in epigenetic modifications of genomic function and functions as disease-specific prognostic biomarkers for lung squamous cell carcinoma (LUSC). This present study aimed to identify methylation-driven genes as prognostic biomarkers for LUSC using bioinformatics analysis.

**Materials and Methods**: Differentially expressed RNAs were obtained using the edge R package from 502 LUSC tissues and 49 adjacent non-LUSC tissues. Differentially methylated genes were obtained using the limma R package from 504 LUSC tissues and 69 adjacent non-LUSC tissues. The methylation-driven genes were obtained using the MethylMix R package from 500 LUSC tissues with matched DNA methylation data and gene expression data and 69 non-LUSC tissues with DNA methylation data. Gene ontology and ConsensusPathDB pathway analysis were performed to analyze the functional enrichment of methylation-driven genes. Univariate and multivariate Cox regression analyses were performed to identify the independent effect of differentially methylated genes for predicting the prognosis of LUSC.

**Results**: A total of 44 methylation-driven genes were obtained. Univariate and multivariate Cox regression analyses showed that twelve aberrant methylated genes (ATP6V0CP3, AGGF1P3, RP11-264L1.4, HIST1H4K, LINC01158, CH17-140K24.1, CTC-523E23.14, ADCYAP1, COX11P1, TRIM58, FOXD4L6, CBLN1) were entered into a Cox predictive model associated with overall survival in LUSC patients. Methylation and gene expression combined survival analysis showed that the survival rate of hypermethylation and low-expression of DQX1 and WDR61 were low. The expression of DQX1 had a significantly negatively correlated with the methylation site cg02034222.

**Conclusion**: Methylation-driven genes DQX1 and WDR61 might be potential biomarkers for predicting the prognosis of LUSC.

## Introduction

Lung cancer is the leading cause of cancer related deaths worldwide 1. Lung squamous cell carcinoma (LUSC) accounts for about 30% of all lung cancers with the highest mortality in the world 2. LUSC accounts for approximately more than 400,000 deaths worldwide each year 3. The five year survival rate of lung squamous cell carcinoma is less than 15% 4. Due to the limitation of treatment and poor survival rate for lung squamous cell carcinoma 5, it is imperative that we explore specific diagnostic and prognostic biomarkers for LUSC. In the present study, we aimed to identify novel specific diagnostic and prognostic biomarkers for predicting survival in LUSC.

Genetic aberrant expression is important for the etiology of human cancer and the combined effect of genetic and epigenetic changes contribute to the progress of human cancer 6-10. DNA methylation is one of the most important elements in epigenetic modifications and participates in the regulation of cellular functions and carcinogenesis 11. Epigenetic modification, especially DNA methylation, plays a significant role in predicting the prognosis of lung cancer 12-15. For instance, the identification of eight DNA methylation biomarkers using high-throughput DNA methylation analysis can predict the prognosis of lung squamous cell carcinoma 16. Aberrant ANK1 methylation contributes to miR-486-5p repression and discriminates lung tumors by histology and smoking status 17. Pharmacologic inhibition of DNA methylation combined with gene expression reveals new specific diagnostic and prognostic biomarkers in lung squamous cell carcinoma 18.

MethylMix is an R package used for identifying disease-specific hyper- and hypo-methylated genes 19. More precisely, MethylMix includes two major characteristics: automatic download of DNA methylation and gene expression data sets from TCGA and automated pre-processing of such data sets: value interpolation, batch correction, and CpG positions within each gene point clustering. Currently, few studies based on using MethylMix R package to identify specific methylation-driven genes have been reported 20-22. Recently, a study based on MethylMix reveals potential prognostic methylation-driven genes for predicting the prognosis in lung adenocarcinoma (LUAD) has been reported 23. In the present study, we extracted the DNA methylation and RNA-Seq data using bioinformatics methods from The Cancer Genome Atlas (TCGA) database, and then the MethylMix R package was performed to obtain methylation-driven genes. Furthermore, a Cox predictive model was established to predict the diagnosis and prognosis of LUSC. Eventually, the joint of methylation and gene expression combined survival analysis was performed to reveal potential specific methylation-driven genes for predicting the prognosis of LUSC.

## Materials and Methods

### Data extraction and analysis

RNA-Seq data and methylation data were downloaded from the Cancer Genome Atlas (TCGA) database. The methylation data consists of 504 LUSC samples and 69 normal samples from the Illumina Infinium Human Methylation 450k platform. The RNA-Seq data (level 3) incorporating lncRNA and mRNA expression was obtained from 502 LUSC samples and 49 normal samples from the IlluminaHiSeq_RNASeq platform. First, based on the limma R package, we retrieved the aberrant methylated genes with the screening criteria absolute fold change (log2) > 0 and adjusting the false discovery rate (FDR) to a *P* value < 0.05. Then, we obtained the differentially expressed lncRNA and mRNA using the edge R package in R software with the absolute fold change (log2) > 0 and adjusted the FDR to a *P* value < 0.05. Next, we used the MethylMix R package with the screening criteria (|logFC|>0, *P*<0.05, Cor<-0.3) to extract the methylation-driven genes. Analysis of average methylation differences at 3000 bp (base pair) sites upstream of genes to identify differential methylation levels in gene promoters 23-25. The differential level of methylation in the promoter of genes was performed by using the limma R package 26. Eventually, we identified methylation-driven genes and aberrant methylated genes to establish a β-mixture model. The data was directly from the TCGA database. No approval was required from the local ethics committee.

### Enrichment analysis of methylation-driven genes in LUSC

We used the Database for Annotation, Visualization and Integrated Discovery (DAVID) (http://david.abcc.ncifcrf.gov/) database to analyze the biological function in methylation-driven genes by using Gene ontology and ConsensusPathDB pathway analysis. In the GO analysis, a *P* value was less than 0.05 was considered as statistically significance. We used the GOCircle and GOChord plotting to explore the relationship between the methylation-driven genes and their biological function. ConsensusPathDB (http://cpdb.molgen.mpg.de/) is an online software incorporating gene regulatory, drug-target interactions and binary complex signaling. In the ConsensusPathDB pathway analysis, *P* < 0.05 was considered as statistically significance.

### Establishment of predictive model of differentially methylated genes based on DNA methylation in LUSC

First, we obtained the expression of differentially methylated genes and retrieved the survival time and survival status of 366 LUSC patients, then used the survival.pl script to extract the data that combined the expression of differentially methylated genes and survival data. Next, we used the univariate R package to obtain the result of univariate Cox regression. We selected 92 aberrant methylated genes to submit to multivariate Cox regression analysis with the screening criteria* P* < 0.05. Based on the median risk score, LUSC patients were divided into two groups, incorporating high-risk groups and low-risk groups. To test the influence on differentially methylated genes signature (high risk vs low risk) on overall survival, we performed the receiver operating characteristic (ROC) curves to calculate the area under the curve (AUC) to reveal prognostic specific biomarkers for predicting survival in LUSC.

### Univariate and multivariate Cox regression independent prognosis analysis of patients of LUSC

In order to further explore the twelve gene signature that can be used as independent prognosis factor, we performed the univariate and multivariate Cox regression independent prognosis analysis of LUSC patients. We extracted 276 LUSC patients with complete clinical information and combined the twelve genes signature risk score of 276 LUSC patients and the expression data of twelve genes of 276 LUSC patients to perform the univariate and multivariate Cox regression independent prognosis analysis. *P* < 0.05 was considered as statistically significant.

### Methylation and gene expression combined survival analysis in LUSC

We used the methylation and gene expression combined survival analysis to further explore the effect of methylation-driven genes on patient prognosis in terms of expression and methylation levels. We performed the joint of methylation and gene expression combined survival analysis to identify potential methylation-driven genes for predicting the prognosis of LUSC patients. Therefore, we performed the Kaplan-Meier curve analysis. *P* < 0.05 was considered as statistically significant.

### Correlation analysis between site methylation and gene expression in LUSC

In order to further explore the relationship between the methylation-driven genes expression and their methylation sites, we used the R package and Perl Package to perform the correlation analysis. First, site methylation data for methylation-driven genes associated with overall survival extracted from the TCGA database using the Perl package, then, we merged site methylation with gene expression data. Finally, we used the R package to figure out the correlation between site methylation and methylation-driven genes expression.

### Survival analysis of methylation site in LUSC patients

In order to further explore the survival rate of methylation site cg02034222 in DQX1 in LUSC patients, we performed the Kaplan-Meier curve analysis of methylation site cg02034222 in DQX1 in LUSC patients. *P* value less than 0.05 was considered as statistically significant.

## Results

### Identification of methylation-driven genes in LUSC

**Figure 1:**
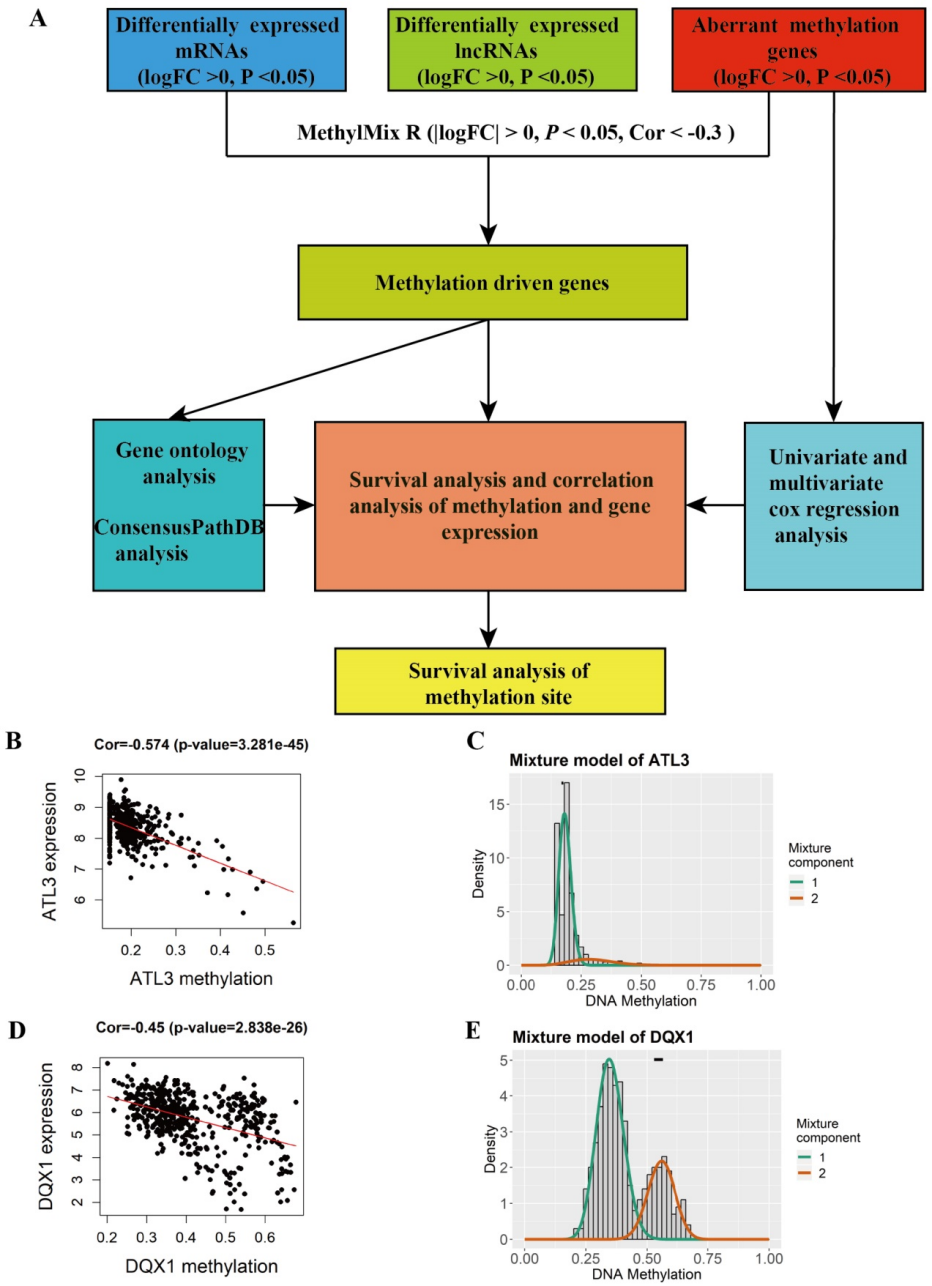
** Identification of methylation and gene expression most relevant genes between LUSC and normal tissue. A** The flow diagram of identification of methylation driven genes in LUSC.** B**, **D** The correlation between methylation and gene expression in methylation driven genes.** C, E** The methylation degree of the methylation-driven genes between LUSC and normal tissue. The red curve indicates the methylation degree from the LUSC tissue, the green curve indicates the methylation degree from the normal tissue. The black line above the figure is the distribution of methylation level in normal tissue.

**Figure 2:**
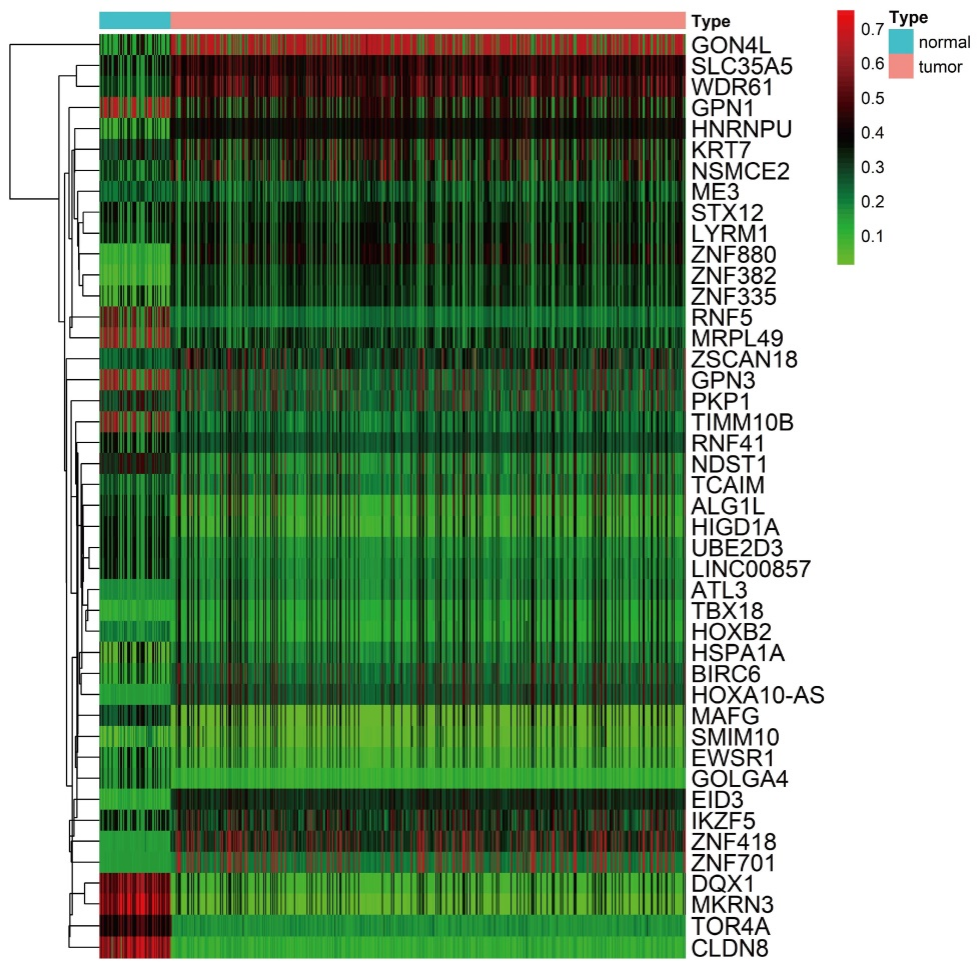
** Heat map of methylation-driven genes between LUSC and normal tissue.** Red represents highly methylated genes and green represents low methylated genes between LUSC and normal tissue.

A total of 44 methylation-driven genes were identified to be connected with DNA methylation using the MethylMix R package. The methylation-driven genes incorporating 42 methylation-driven mRNAs and two methylation-driven lncRNAs were shown in Table 1. Figure 1B and 1D shows methylation-driven gene ATL3 and DQX1 have significant negative correlation in methylation and gene expression level. The distribution of the methylation degree shows that ATL3 is hyper-methylated in LUSC patients and hypo-methylated in normal patients (Figure 1C). The distribution of the methylation degree shows that DQX1 is hyper-methylated in non-LUSC patients and hypo-methylated in LUSC patients (Figure 1E). A flow diagram of methylation-driven genes is shown in Figure 1A. A heat-map of methylation driven genes mRNAs and lncRNAs is shown in Figure 2.

### Functional enrichment analysis of methylation-driven genes in LUSC

**Figure 3:**
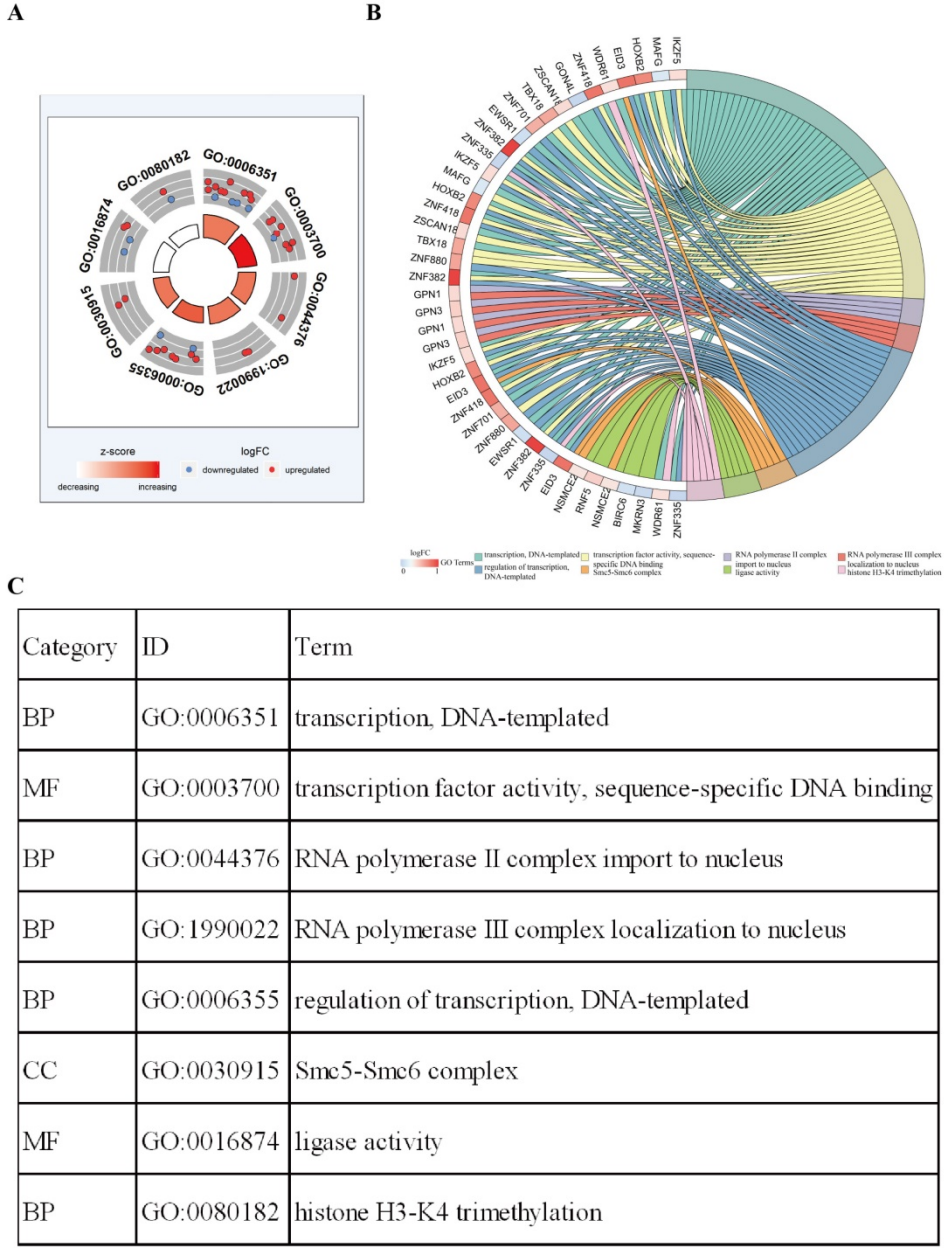
** Functional enrichment analysis of methylation-driven genes in LUSC. A** The outer circle represents the expression of methylation driven mRNAs in each enriched GO terms: red dots which were on each GO terms indicated the up-regulated methylation driven mRNAs, the inner circle indicates the significance of GO terms (log10-adjusted *P* values). Blue dots indicate the down-regulated methylation driven mRNAs. **B** The circle represents the correlation between 42 methylation driven mRNAs and their GO terms. **C** The distribution of methylation driven mRNAs in significant GO terms.

**Figure 4:**
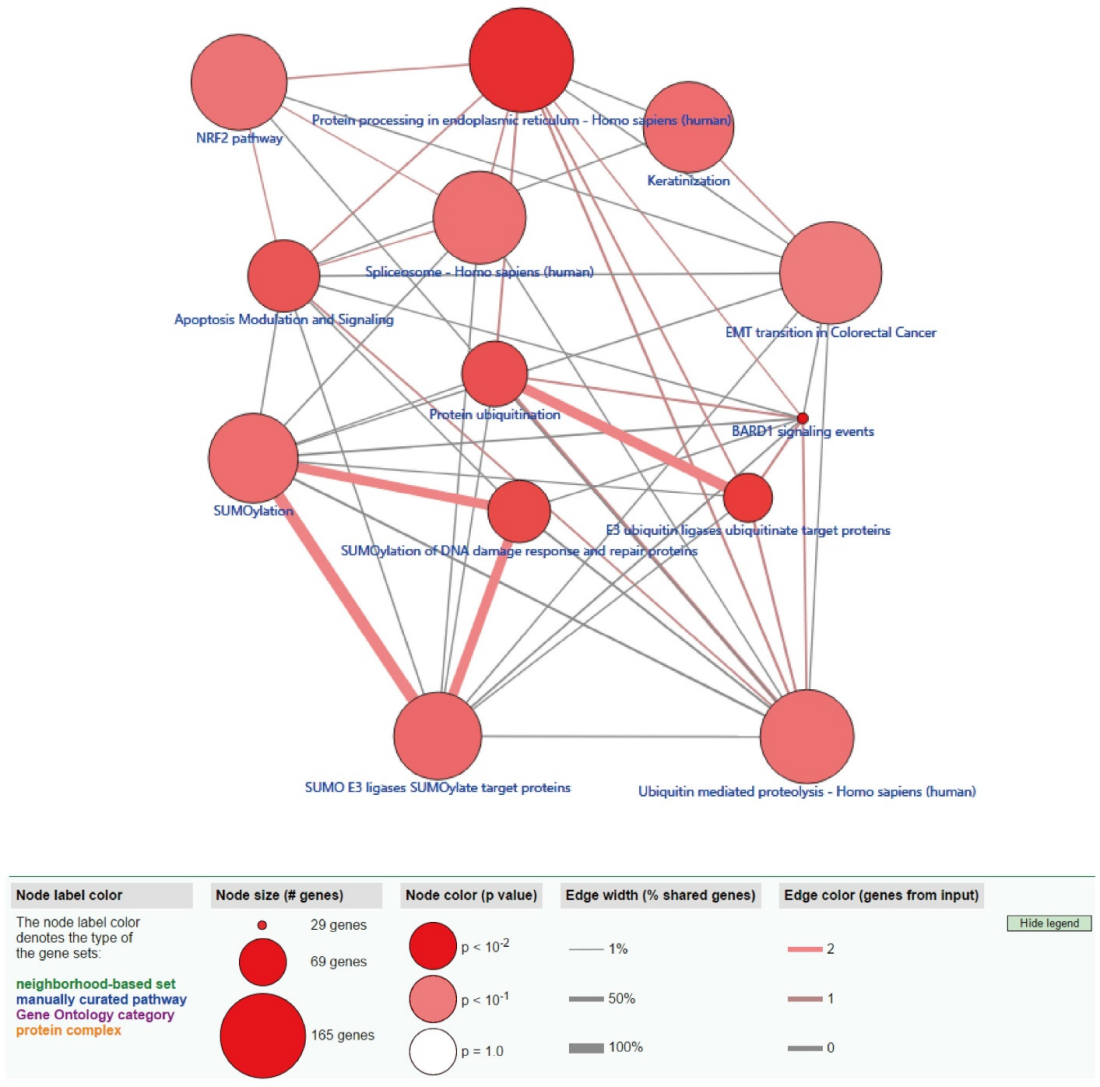
** ConsensusPathDB pathway analysis of methylation-driven genes in LUSC.** The red circles indicate the number of methylation driven genes on each pathway. The line between the two red circles indicates the ratio of methylation driven genes present in the common genes of the two pathways.

Functional enrichment analysis shows that eight GO terms (transcription, DNA-templated; transcription factor activity, sequence-specific DNA binding; RNA polymerase II complex import to nucleus; RNA polymerase III complex localization to nucleus; regulation of transcription, DNA-templated; Smc5-Smc6 complex; ligase activity; histone H3-K4 tri-methylation) with statistical significance (*P* < 0.05). The highest GO term was biological process “GO0006351 transcription, DNA templated” (Figure 3A and 3C). Figure 3B shows the all methylation-driven mRNAs with their related eight GO terms. Figure 4 shows that 13 pathways (BARD1 signaling events; Protein processing in endoplasmic reticulum - Homo sapiens (human); E3 ubiquitin ligases ubiquitinate target proteins; SUMOylation of DNA damage response and repair proteins; Protein ubiquitination; Apoptosis Modulation and Signaling; SUMO E3 ligases SUMOylate target proteins; SUMOylation; Keratinization; Spliceosome - Homo sapiens (human); Ubiquitin mediated proteolysis - Homo sapiens (human); NRF2 pathway; EMT transition in Colorectal Cancer) were considered statistically significant (*P* < 0.05). As we can see from the Figure 4, the methylation-driven genes were most enriched in BARD1 signaling events, Protein processing in endoplasmic reticulum - Homo sapiens (human) and E3 ubiquitin ligases ubiquitinate target proteins (*P*<0.01).

The pathway analysis is shown in Table 2.

### Establishment of predictive model of 12 differentially methylated genes associated with overall survival in LUSC

Univariate Cox regression analysis was first used to identify differentially methylated genes associated with overall survival in LUSC; incorporating 92 differentially methylated genes were selected to submit to perform multivariate Cox regression analysis. *(P* < 0.05) Multivariate Cox regression analysis shows that 12 differentially methylated genes were finally selected to establish a predictive model. The linear combination of the expression of 12 aberrant methylated genes was performed to establish the predictive model. The relative coefficients weighted in the multivariate Cox regression are as follows: survival risk score = ((-1.7055) × expression value of ATP6V0CP3 + (-2.4550) × expression value of AGGF1P3 + (-2.0585) × expression value of RP11-264L1.4 + 3.1730 × expression value of HIST1H4K + 4.3379 × expression value of LINC01158 + 1.2379 × expression value of CH17-140K24.1 + (-2.3096) × expression value of CTC-523E23.14 + (-3.5519) × expression value of ADCYAP1 + (-1.1394) × expression value of COX11P1 + (-2.0323) ×expression value of TRIM58 + (-1.8202) × expression value of FOXD4L6 + 3.9318 × expression value of CBLN1). The multivariate Cox regression analysis is shown in Table 3.

### ROC curve analysis and risk groupings

**Figure 5:**
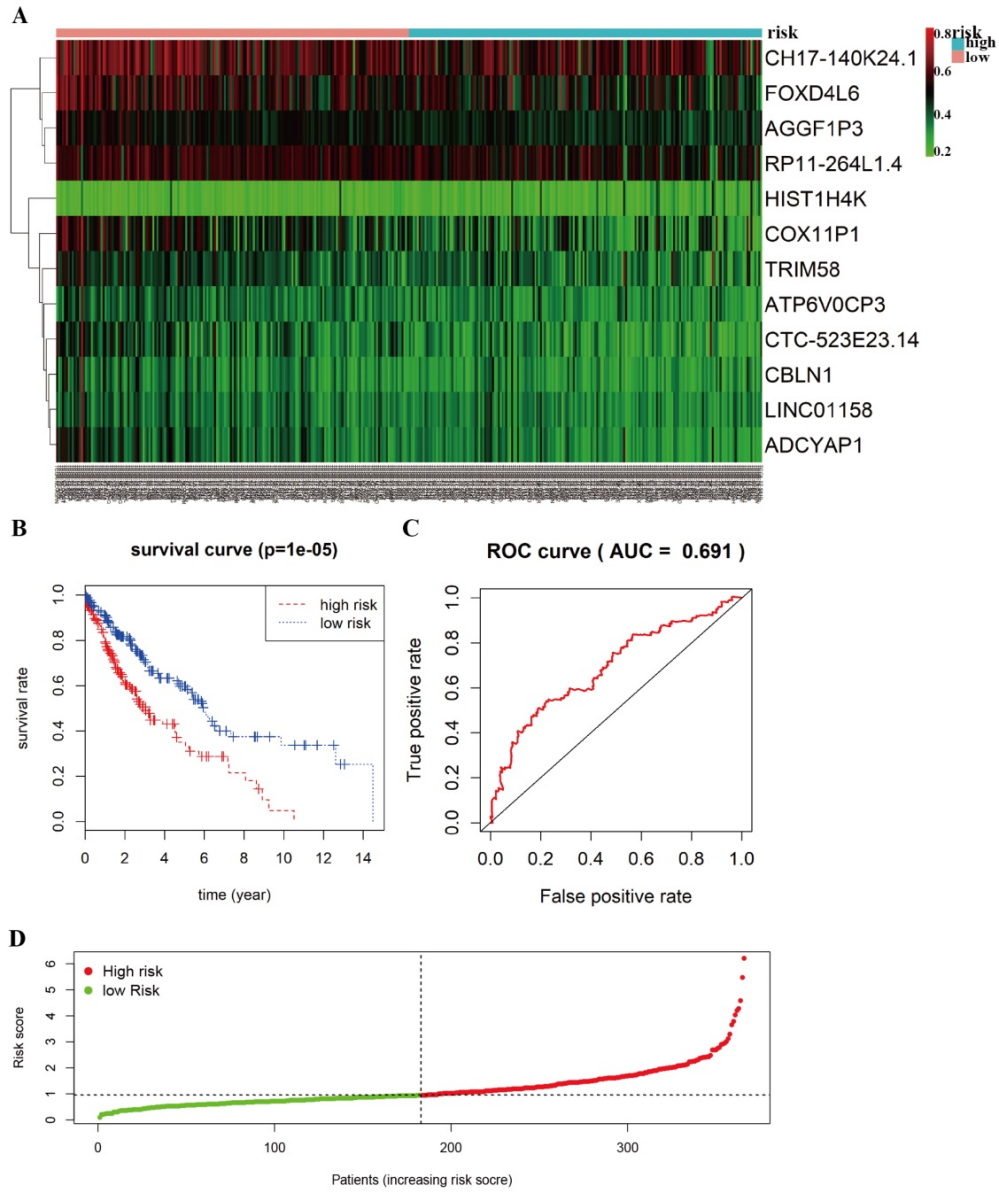
** Prognostic value of 12-differentially methylated genes based on DNA methylation in LUSC. A** Risk heat-map predictive model established from 12 differentially methylated genes from 366 LUSC patients. **B** Kaplan-Meier curve analysis of 12 differentially methylated genes for overall survival of LUSC patients. **C** ROC curve analysis of prognostic 12 differentially methylated genes signature. **D** The riskscore in the Cox model between high risk group and low risk group of LUSC.

The heat-map shows that 12 differentially methylated genes (ATP6V0CP3, AGGF1P3, RP11-264L1.4, HIST1H4K, LINC01158, CH17-140K24.1, CTC-523E23.14, ADCYAP1, COX11P1, TRIM58, FOXD4L6, CBLN1) were divided into two groups based on the median risk scores (Figure 5A). A total of 366 patients with complete survival information were divided into a high-risk group (n=183) and a low-risk group (n=183). The Kaplan-Meier curve with a Log-rank statistical examination was used to perform survival analysis (Figure 5B). As shown in Figure 5B, patients in the high-risk group had significantly poor survival rate than in the low-risk group (*P* =1e-05). Receiver operating characteristic (ROC) curve was performed to identify the effect of 12 aberrant methylated genes signature associated with overall survival in LUSC (Figure 5C). The graph of the risk score between high risk group and low risk group in the Cox model is shown in Figure 5D.

### Univariate and multivariate Cox regression analysis for the various DNA methylation classifiers of patients with LUSC

**Figure 6:**
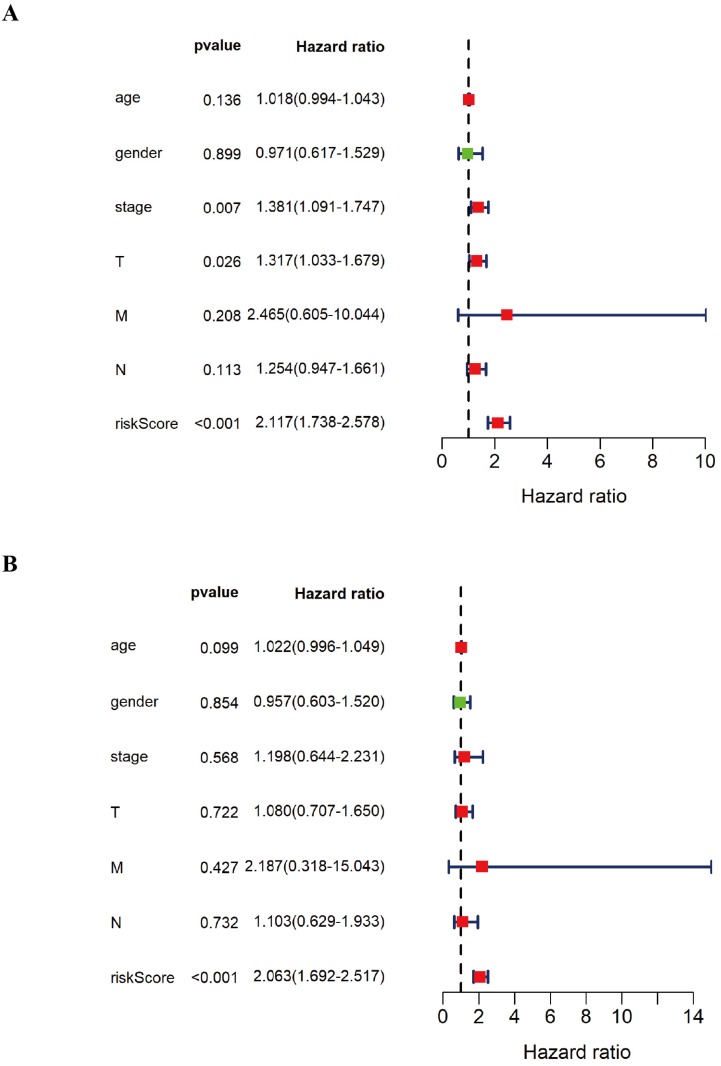
** The forest plot of univariate and multivariate Cox regression analysis of the various DNA methylation classifiers of LUSC patients. A** The forest plot of univariate Cox regression independent prognosis analysis of LUSC patients. **B** The forest plot of multivariate Cox regression independent prognosis analysis of LUSC patients. The red mark in the plot indicates that this clinical feature is a high risk factor. The green mark in the plot indicates that this clinical feature is a low risk factor.

In order to further verify the twelve gene signature is an independent prognosis factor, we used the univariate and multivariate Cox regression analysis. We extracted the complete LUSC clinical features with 276 samples and then combined the twelve gene expression data and the prognostic risk score with the LUSC clinical features, including LUSC age, gender, pathology stage, pathology T stage, pathology M stage, pathology N stage to perform the univariate and multivariate Cox regression analysis. The forest plot of univariate and multivariate Cox regression analysis was showed in Figure 6. Univariate Cox regression analysis showed that pathology stage, pathology T stage and twelve gene signature risk score can act as independent prognostic factor (Figure 6A). Multivariate Cox regression analysis showed that twelve gene signature is an independent prognosis factor (Figure 6B) (Table 4).

### Methylation and gene expression combined survival analysis in LUSC

**Figure 7:**
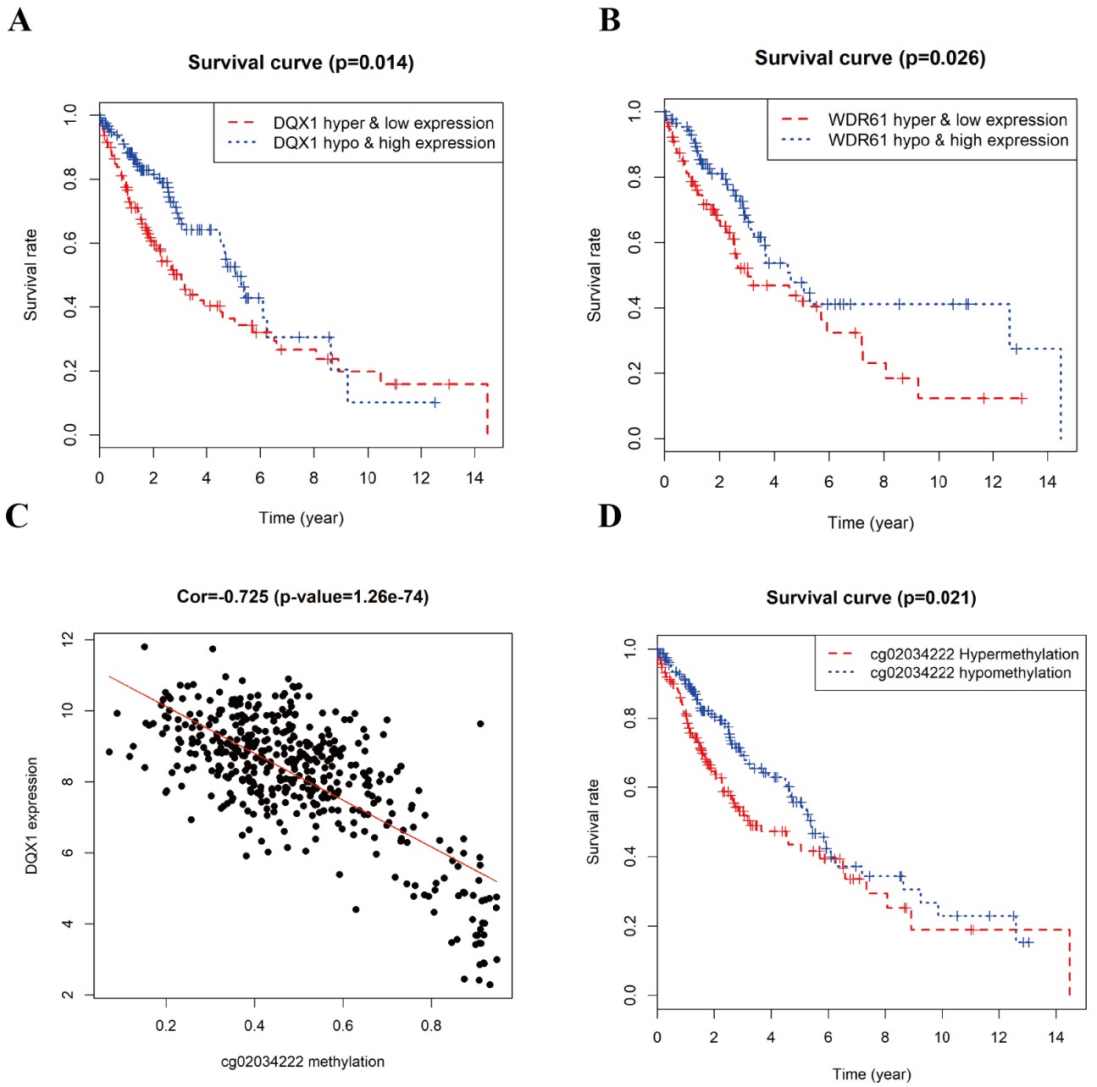
** Survival analysis and correlation analysis of methylation and gene expression and survival analysis of methylation site of LUSC. A, B** Kaplan-Meier curve analysis of the joint of DNA methylation and gene expression in LUSC patients. **C** The correlation analysis between the expression of DQX1 and cg02034222 methylation in LUSC patients. **D** Kaplan-Meier curve analysis of methylation site cg02034222 in DQX1 in LUSC patients.

The combined survival analysis revealed that the joint of methylation and gene expression of the genes (DQX1, WDR61) had significant correlation with the prognosis of LUSC patients (Figure 7A and 7B). As shown in Figure 6, the hypermethylation and low-expression survival rate of DQX1 and WDR61 were low. The joint of methylation and gene expression survival analysis shows that the DNA methylation and gene expression of DQX1 and WDR61 were associated with overall survival in LUSC patients (*P* < 0.05). We used the median to define the DNA methylation cut-off to classify a sample as hypermethylated or hypomethylated about the survival rate based on DQX1 and WDR61 data.

### Correlation analysis between gene expression and methylation sites

To figure out the relationship between the DNA methylation and gene expression level in DQX1 and WDR61, we used the Perl package to obtain the methylation sites in DQX1 and WDR61, the expression of DQX1 had 8 methylation sites in TCGA database, while only one methylation site had significant correlation between expression and DNA methylation level. The expression of WDR61 had two methylation sites in TCGA database, while no methylation site had significant correlation with the expression of WDR61. As is shown in Figure 7C, the expression of DQX1 had a significant correlation (Cor = -0.725) with cg02034222 methylation (*P* = 1.26e-74).

### Survival analysis of methylation site cg02034222 in DQX1 in LUSC patients

In order to further figure out whether the one methylation site (cg02034222) in DQX1 is responsible of the survival rate of LUSC patients, we performed survival analysis of methylation site (cg02034222) in DQX1 in hypermethylation and hypomethylation LUSC patients (Figure 7D). The one CpG site cg02034222 in DQX1 in hypermethylation LUSC patients had a poor survival rate than the one CpG site cg02034222 in DQX1 in hypomethylation LUSC patients (*P* = 0.021).

## Discussion

In recent years, with the increasing numbers of advanced diagnosis and poor prognosis in lung squamous cell carcinoma, it is crucial to explore more effective diagnostic and prognostic biomarkers for predicting survival in LUSC. Genetic and epigenetic changes facilitate the progression of LUSC. DNA methylation and RNA-Seq data analysis provide a novel perspective to reveal disease specific diagnostic and prognostic biomarkers in human lung cancer 27,28. The rapid development of RNA-Seq analysis technologies provides a novel perspective to explore the molecular characteristic and pathogenesis of LUSC and provides significant evidence for predicting the prognosis of LUSC. Emerging evidences shows that the studies on the molecular mechanism of LUSC and the prognostic biomarkers of the LUSC associated with methylation driven genes is still lacking. In our study, we used the MethylMix R package to identify methylation driven genes and a cox predictive model was established to predict the prognosis of LUSC and the joint of methylation and gene expression combined survival analysis reveals potential prognostic biomarkers for predicting the prognosis of LUSC.

Epigenetics modification, especially DNA methylation, participates in the pathogenesis of LUSC. Accumulating evidences have demonstrated that DNA methylation acts as the major molecular mechanism of epigenetic modification was associated with the human malignant cancer, incorporating lung cancer 29-31. The joint of gene and DNA methylation using bioinformatics analysis revealing new diagnostic and prognostic biomarkers for predicting the prognosis of cancer 32-34. Therefore, it is pivotal to identify disease-specific prognostics biomarkers to determine the exploration of the molecular mechanism of LUSC. The methylation of L1RE1, RARB, and RASSF1 acts as potential disease specific biomarkers for predicting the prognosis of lung cancer 35. TRIM58/cg26157385 methylation associated with eight genes including A2ML1, CCNE1, COBL, ESCO2, GPR115, MMP10, OVOL1 and SCGB1A1 in lung squamous cell carcinoma 36. The prognostic value of HOXA9 promoter methylation was associated with lung cancer and can become a new diagnostic and prognostic biomarker for predicting the prognosis of stage I lung adenocarcinoma 37. Therefore, bioinformatics analysis of DNA methylation and gene expression provide a significant horizon for identifying disease specific diagnostic and prognostic biomarkers in lung squamous cell carcinoma.

Functional enrichment analysis of methylation-driven mRNA reveals the methylation-driven genes might be mostly associated with the biologic function of transcription, DNA template; transcription factor activity; RNA polymerase II complex import to nucleus (*P* < 0.05). The pathway analysis shows that the methylation-driven genes were associated with 13 pathways (*P* < 0.05); the most enriched pathway was the Protein processing in endoplasmic reticulum-Homo sapiens (human).

In our study, univariate and multivariate Cox regression analysis shows that ATP6V0CP3, AGGF1P3, RP11-264L1.4, HIST1H4K, LINC01158, CH17-140K24.1, CTC-523E23.14, ADCYAP1, COX11P1, TRIM58, FOXD4L6, CBLN1 were associated with overall survival and could establish a survival predictive model to predict the prognosis of LUSC. The 12 differentially methylated genes could also act as an independent prognostic factor for predicting the prognosis of LUSC. HIST1H4K can be used as prognostic factors for predicting the prognosis of cervical cancer patients 38. HIST1H4K can be used as DNA methylation biomarkers for predicting the prognosis of prostate cancer 39. ADCYAP1 can act as prognostic biomarkers to predict risk of endometrial cancer 40. Recently a study based on TCGA database reveal ADCYAP1 can act as prognostic biomarkers for predicting the prognosis of LUSC 4. ADCYAP1 can act as novel biomarkers for predicting the prognosis of cervical precancer and cancer 41. TRIM58/cg26157385 methylation may play a significant role for predicting the prognosis of LUSC 36. CBLN1 can be used as marker genes for predicting the prognosis of the Ventromedial Hypothalamic Nucleus 42.

In our study, the joint of methylation and gene expression combined survival analysis shows that the survival rate of hypermethylation and low-expression of DQX1 was significant lower than the survival rate of hypomethylation and high-expression of DQX1. Epigenome-wide association studies (EWASs) suggested a novel association between T2D and cg06721411(DQX1; P value=1.18×10(-9)) 43. The survival rate of hypomethylation and high-expression of WDR61 was significant higher than the survival rate of hypermethylation and low-expression of WDR61. Furthermore, we performed the correlation analysis between DNA methylation sites and gene expression. The expression of DQX1 was significantly negatively associated with the methylation sites cg02034222 (*P* value= 1.26e-74), which might provide a significant horizon to explore prognostic biomarkers for predicting the diagnosis and prognosis of LUSC. Compared with previous studies^4^, our study first obtained aberrant methylated genes using the limma R package, obtained differentially expressed genes using the edge R package and then we filtered the missing values and low expression genes and intersected gene expression data with DNA methylation data to input MethylMix R package to identify methylation driven genes, which may provide a novel perspective to reveal disease-specific prognostic biomarkers in LUSC and may play a significant role in predicting the diagnosis and prognosis of LUSC.

## Conclusions

In our study, we aimed to identify methylation-driven genes using MethylMix between LUSC patients and normal samples from the TCGA database for predicting the prognosis of lung squamous cell carcinoma. Univariate and multivariate Cox regression analysis showed that the survival predictive model established by twelve differentially methylated genes (ATP6V0CP3, AGGF1P3, RP11-264L1.4, HIST1H4K, LINC01158, CH17-140K24.1, CTC-523E23.14, ADCYAP1, COX11P1, TRIM58, FOXD4L6, CBLN1) can act as independent prognostic factor for predicting the prognosis of LUSC. In addition, the DNA methylation and gene expression levels of DQX1 and WDR61 are significantly associated with overall survival and the expression of DQX1 has a significantly negatively correlated with the methylation site cg02034222. Our study may provide a novel perspective for predicting the prognosis of LUSC.

## Figures and Tables

**Table 1:** Methylation-driven mRNAs and lncRNAs in LUSC

Gene	Normal Mean	Tumor Mean	logFC	*P*-Value	Adjusted-*P*	cor	Cor *P*-Value
ATL3	0.17248799	0.19693593	0.19123	2.67E-05	0.001522	-0.5742885	3.28E-45
EID3	0.11947906	0.22283156	0.899196	1.50E-06	8.54E-05	-0.557997212	2.91E-42
HSPA1A	0.17681654	0.17435814	-0.0202	0.000498	0.028362	-0.546760366	2.54E-40
KRT7	0.32703655	0.40270931	0.300287	5.94E-08	3.39E-06	-0.535706329	1.75E-38
ALG1L	0.26423898	0.14912111	-0.82536	2.11E-20	1.20E-18	-0.522826399	2.01E-36
HOXB2	0.19379899	0.32946771	0.765576	2.62E-07	1.49E-05	-0.49642396	1.81E-32
ZNF701	0.1511366	0.21621834	0.516636	2.00E-19	1.14E-17	-0.495379361	2.55E-32
SMIM10	0.11304237	0.20223432	0.839164	1.07E-05	0.000613	-0.453929124	8.73E-27
DQX1	0.5466067	0.41245817	-0.40626	1.12E-17	6.41E-16	-0.449792303	2.84E-26
ZNF382	0.09030249	0.20544586	1.18592	1.78E-15	1.01E-13	-0.449122352	3.43E-26
MKRN3	0.59970621	0.46968784	-0.35255	4.32E-12	2.46E-10	-0.440157315	4.15E-25
RNF41	0.3089854	0.3720367	0.267906	3.83E-08	2.18E-06	-0.426198823	1.75E-23
TBX18	0.12078327	0.17927841	0.569781	1.55E-07	8.83E-06	-0.413942368	4.04E-22
MRPL49	0.4402478	0.53150492	0.271767	1.10E-07	6.27E-06	-0.409474247	1.23E-21
HIGD1A	0.28409562	0.35668444	0.328272	4.55E-10	2.59E-08	-0.407902793	1.81E-21
ZNF418	0.14960837	0.27538919	0.880281	2.27E-20	1.29E-18	-0.403101005	5.86E-21
EWSR1	0.23855847	0.19310873	-0.30493	1.42E-07	8.07E-06	-0.402188496	7.30E-21
GOLGA4	0.23063898	0.20194077	-0.1917	0.000172	0.009832	-0.396381517	2.93E-20
ZSCAN18	0.24775191	0.29098815	0.232064	5.04E-08	2.87E-06	-0.39602996	3.18E-20
ME3	0.24575626	0.31586615	0.362085	8.19E-12	4.67E-10	-0.390298349	1.22E-19
TOR4A	0.43481132	0.50342309	0.211382	1.19E-12	6.78E-11	-0.386093997	3.20E-19
ZNF335	0.17544474	0.13484788	-0.37968	2.95E-05	0.001681	-0.385838776	3.39E-19
GON4L	0.24833952	0.18937769	-0.39105	1.24E-13	7.09E-12	-0.378884895	1.63E-18
GPN1	0.44840968	0.53171848	0.245845	0.000366	0.020889	-0.3688374	1.47E-17
NDST1	0.37244227	0.39375627	0.080286	3.03E-05	0.001725	-0.367888744	1.80E-17
LYRM1	0.26940325	0.33139827	0.298799	1.71E-10	9.73E-09	-0.363619695	4.48E-17
ZNF880	0.10955173	0.16412283	0.583164	3.15E-08	1.80E-06	-0.358551732	1.29E-16
HNRNPU	0.18185654	0.14481692	-0.32857	3.32E-08	1.89E-06	-0.355947775	2.22E-16
PKP1	0.33730017	0.28220385	-0.2573	1.19E-09	6.79E-08	-0.355519119	2.42E-16
CLDN8	0.59729707	0.5081929	-0.23307	1.40E-07	7.95E-06	-0.353071085	3.99E-16
GPN3	0.44053766	0.54189041	0.298736	1.93E-10	1.10E-08	-0.351039605	6.03E-16
STX12	0.29864643	0.38069839	0.35021	1.20E-10	6.82E-09	-0.350277807	7.03E-16
MAFG	0.30194995	0.26400157	-0.19376	4.30E-06	0.000245	-0.347374761	1.26E-15
BIRC6	0.18005362	0.14959829	-0.26733	0.000359	0.020475	-0.343441891	2.74E-15
NSMCE2	0.25383424	0.28660713	0.175188	0.00031	0.017663	-0.342286449	3.44E-15
IKZF5	0.27924672	0.32474861	0.217783	6.03E-05	0.00344	-0.336552652	1.05E-14
TIMM10B	0.41801039	0.51747519	0.307951	8.21E-10	4.68E-08	-0.33036822	3.38E-14
SLC35A5	0.30500029	0.36714383	0.267535	2.83E-08	1.61E-06	-0.326827649	6.55E-14
WDR61	0.25539793	0.29489636	0.207461	1.50E-06	8.55E-05	-0.326478841	6.98E-14
TCAIM	0.23709962	0.29466613	0.313588	1.15E-10	6.54E-09	-0.323459542	1.22E-13
RNF5	0.39135838	0.48335106	0.304581	1.75E-09	9.96E-08	-0.31250345	8.67E-13
UBE2D3	0.29164547	0.34557935	0.244801	2.00E-06	0.000114	-0.309087728	1.57E-12
HOXA10-AS	0.14920717	0.24212565	0.698439	2.43E-13	9.74E-13	-0.343245543	2.85E-15
LINC00857	0.28641727	0.34963067	0.287713	0.0046	0.018401	-0.512263266	8.45E-35

**Table 2:** Pathway analysis

Pathway	count	*P*-value	q-value
BARD1 signaling events	2	0.00166	0.05648
Protein processing in endoplasmic reticulum - Homo sapiens (human)	3	0.00483	0.07633
E3 ubiquitin ligases ubiquitinate target proteins	2	0.00674	0.07633
SUMOylation of DNA damage response and repair proteins	2	0.01098	0.08233
Protein ubiquitination	2	0.01211	0.08233
Apoptosis Modulation and Signaling	2	0.01547	0.08766
SUMO E3 ligases SUMOylate target proteins	2	0.02599	0.10038
SUMOylation	2	0.02844	0.10038
Keratinization	2	0.02928	0.10038
Spliceosome - Homo sapiens (human)	2	0.03186	0.10038
Ubiquitin mediated proteolysis - Homo sapiens (human)	2	0.03318	0.10038
NRF2 pathway	2	0.03543	0.10038
EMT transition in Colorectal Cancer	2	0.04399	0.11506

**Table 3:** Multivariate Cox regression analysis of 12 genes associated with overall survival in LUSC patients

	coef	exp(coef)	se(coef)	z	*p*
ATP6V0CP3	-1.7055	0.1817	1.06	-1.61	0.108
AGGF1P3	-2.455	0.0859	1.586	-1.55	0.122
RP11-264L1.4	-2.0585	0.1276	1.034	-1.99	0.046
HIST1H4K	3.173	23.8791	1.1964	2.65	0.008
LINC01158	4.3379	76.5481	2.1068	2.06	0.039
CH17-140K24.1	1.2379	3.4482	0.8287	1.49	0.135
CTC-523E23.14	-2.3096	0.0993	1.1061	-2.09	0.037
ADCYAP1	-3.5519	0.0287	1.6107	-2.21	0.027
COX11P1	-1.1394	0.32	0.7318	-1.56	0.119
TRIM58	-2.0323	0.131	0.9696	-2.1	0.036
FOXD4L6	-1.8202	0.162	0.8431	-2.16	0.031
CBLN1	3.9318	51.0005	1.9069	2.06	0.039

**Table 4:** Univariate and multivariate Cox regression analysis of LUSC clinical characteristics based on predictive model

Variables	Univariate analysis		Multivariate analysis	
	Hazards ratio (95% CI)	*P* value	Hazards ratio (95% CI)	*P* value
Age	1.018 (0.994-1.043)	0.136	1.022 (0.996-1.049)	0.099
Gender	0.971 (0.617-1.529)	0.899	0.957 (0.603-1.520)	0.854
Stage	1.381 (1.091-1.747)	0.007	1.198 (0.644-2.231)	0.568
T	1.317 (1.033-1.679)	0.026	1.080 (0.707-1.650)	0.722
M	2.465 (0.605-10.044)	0.208	2.187 (0.318-15.043)	0.427
N	1.254 (0.947-1.661)	0.113	1.103 (0.629-1.933)	0.732
riskScore	2.117 (1.738-2.578)	<0.001	2.063 (1.692-2.517)	<0.001

## Abbreviations

AUC: area under the curve
DAVID: the database for annotation, visualization and integrated discovery
EWASs: epigenome-wide association studies
FC: fold change
FDR: false discovery rate
GO: gene ontology
LUSC: lung squamous cell carcinoma
ROC: receiver operating characteristic
TCGA: the cancer genome atlas
